# Anomalous Origin of the Left Main Coronary Artery From the Proximal Right Coronary Artery With a Benign Prepulmonic Course: A Rare Single Coronary Artery Variant

**DOI:** 10.7759/cureus.111114

**Published:** 2026-06-18

**Authors:** Jyothsna Goranti, Sachin Sapkota, Abirami Rajendiran, Azucena Del Real, Mehran Abolbashari

**Affiliations:** 1 Internal Medicine, The Hospitals of Providence-Transmountain/Texas Tech University Health Sciences Center El Paso, El Paso, USA; 2 Cardiology, Center of the Heart, EL Paso, USA

**Keywords:** anomalous coronary origin, coronary artery anomaly, coronary ct angiography, left main coronary artery, prepulmonic course, single coronary artery, sudden cardiac death

## Abstract

Coronary artery anomalies (CAAs) are rare congenital variations with a broad spectrum of clinical implications. While certain configurations are associated with myocardial ischemia and sudden cardiac death (SCD), many remain clinically silent and are discovered incidentally. An anomalous origin of the left main coronary artery (LMCA) from the right coronary artery (RCA) represents an exceptionally uncommon variant, particularly when it arises from the proximal RCA and follows a prepulmonic course.

We report the case of a 56-year-old female with a history of immune thrombocytopenic purpura who presented with atypical chest discomfort. She underwent coronary computed tomography angiography (CCTA), which revealed an LMCA originating from the proximal RCA with a prepulmonic (anterior) course, consistent with a Lipton right sinus of valsalva, type II, anterior (RIIA) single coronary artery (SCA) pattern. The coronary artery calcium score was zero, and no evidence of obstructive coronary artery disease was identified. Given the absence of ischemia and the benign anatomical course, the patient was managed conservatively. She underwent periodic outpatient surveillance with symptom reassessment and cardiovascular risk factor monitoring, remaining asymptomatic over three years without adverse cardiac events.

Single coronary artery anomalies are rare, with reported prevalence estimates of approximately 0.06% in angiographic series, although these data may be influenced by referral bias. Among these, LMCA arising from the RCA accounts for only 0.0024%-0.02% of reported cases. Clinical significance depends on vessel course as well as associated morphological features. Interarterial variants carry higher risk, whereas prepulmonic and retroaortic courses are generally considered lower risk. CCTA is valuable for defining anatomy and guiding individualized risk stratification, while functional testing may be considered selectively when symptoms or uncertain hemodynamic significance are present.

This case highlights a rare but benign coronary anomaly and underscores the importance of integrating anatomical findings, clinical presentation, and selective adjunctive testing to guide management while avoiding unnecessary invasive intervention.

## Introduction

Coronary artery anomalies (CAAs) comprise a diverse group of congenital variations affecting the origin, trajectory, or structural anatomy of the coronary vasculature. Their reported prevalence ranges from approximately 0.2% to 1.3% in patients undergoing coronary angiography and about 0.3% in autopsy-based studies [1,2]. Although many of these anomalies are asymptomatic and detected incidentally, certain patterns have been linked to myocardial ischemia and an increased risk of sudden cardiac death (SCD), particularly among younger individuals and athletes engaged in competitive sports [3,4].

Single coronary artery (SCA) anomalies represent a particularly uncommon subset, with an estimated incidence of approximately 0.06% in large angiographic series [5]. Within this group, the anomalous origin of the left main coronary artery (LMCA) from the right coronary sinus or right coronary artery (RCA) is exceedingly rare, with reported prevalence between 0.0024% and 0.02% [1,5]. Observational imaging studies have further highlighted the overall low frequency of congenital coronary abnormalities in the general adult population [6].

The clinical implications of CAAs are largely determined by the anatomical pathway of the anomalous vessel rather than its origin alone. Variants with an interarterial course, particularly those traversing between the aorta and pulmonary artery, have been associated with a higher likelihood of ischemia and SCD. This increased risk has been attributed to factors such as a narrowed or slit-like ostium, acute angulation at the origin, intramural segments within the aortic wall, and dynamic compression during periods of increased cardiac demand [3,7]. In contrast, anomalies with prepulmonic (anterior), retroaortic, or transseptal courses are generally considered low-risk and are often associated with favorable clinical outcomes [8,9].

Advances in noninvasive cardiac imaging have significantly improved the evaluation of coronary anomalies. Coronary computed tomography angiography (CCTA) offers high-resolution three-dimensional visualization of coronary origin, proximal course, and surrounding anatomical relationships and may identify anomalies more accurately than conventional invasive angiography [10,11]. However, while CCTA is highly effective for anatomical definition, it may not fully determine the functional significance of an anomaly during peak physiological stress. In selected patients with exertional symptoms or uncertain hemodynamic relevance, adjunctive stress imaging or provocative functional testing may provide additional clinical value.

Despite improved anatomical characterization, longitudinal management of lower-risk coronary anomaly variants remains less clearly defined. At present, universally standardized international surveillance recommendations are limited, and follow-up strategies are often individualized according to symptom burden, anatomical features, patient age, and associated cardiovascular risk factors.

In this report, we describe a rare case of an anomalous LMCA arising from the proximal RCA with a prepulmonic course, identified incidentally on CCTA and managed conservatively with a favorable long-term clinical outcome.

## Case presentation

A 56-year-old female with a medical history significant for immune thrombocytopenic purpura (ITP) presented with intermittent atypical chest discomfort. The episodes were non-exertional, transient, and not associated with dyspnea, diaphoresis, syncope, palpitations, nausea, or radiation of pain. She denied exercise intolerance or worsening symptoms with activity. Physical examination was unremarkable. Baseline electrocardiogram and cardiac biomarkers were normal.

On presentation, vital signs were stable: temperature was 36.7°C, heart rate was 73 bpm, respiratory rate was 18 breaths/min, blood pressure was 117/72 mmHg, and oxygen saturation was 99% on room air. Cardiopulmonary examination revealed a regular heart rhythm without murmurs and clear lungs with non-labored respirations. The ECG revealed normal sinus rhythm. Initial laboratory evaluation demonstrated thrombocytopenia and mild biochemical abnormalities, with otherwise unremarkable cardiac biomarkers and renal function (Table 1). Mild transaminase elevation was noted without hyperbilirubinemia or clinical evidence of hepatic dysfunction and was considered incidental to the presenting complaint.

**Table 1:** Laboratory Results on Admission. WBC: white blood cell count; AST: aspartate aminotransferase; ALT: alanine aminotransferase; HDL: high-density lipoprotein; LDL: low-density lipoprotein; BNP: B-type natriuretic peptide; TSH: thyroid-stimulating hormone.

Laboratory Test	Patient Value	Reference Range (Units)
White blood cell count (WBC)	5.8	4.0–11.0 ×10³/µL
Hemoglobin	12.7	13.5–17.5 g/dL
Platelet count	34	150–400 ×10³/µL
Sodium	140	135–145 mmol/L
Potassium	3.4	3.5–5.0 mmol/L
Creatinine	0.6	0.7–1.3 mg/dL
Aspartate aminotransferase (AST)	71	10–40 U/L
Alanine aminotransferase (ALT)	59	7–56 U/L
Total bilirubin	0.30	0.1–1.2 mg/dL
Hemoglobin A1c	6.2	<5.7 %
Total cholesterol	176	<200 mg/dL
HDL cholesterol	40.1	>40 mg/dL
LDL cholesterol (calculated)	108.3	<100 mg/dL
Triglycerides	138	<150 mg/dL
B-type natriuretic peptide (BNP)	13	<100 pg/mL
Troponin I	2.4–2.5	8.7–18.7 pg/mL
Thyroid-stimulating hormone (TSH)	8.94	0.4–4.0 µIU/mL
Free T4	0.81	0.8–1.8 ng/dL

Given persistent symptoms and the need to exclude clinically significant coronary pathology, coronary computed tomography angiography (CCTA) was performed. The patient’s history of ITP with thrombocytopenia was also considered during diagnostic planning, favoring an initial non-invasive imaging strategy given the potential bleeding risk associated with invasive coronary angiography. Imaging revealed an anomalous origin of the LMCA arising from the proximal RCA (Figure 1). The anomalous LMCA followed a prepulmonic (anterior) course before bifurcating into the left anterior descending and left circumflex arteries. There was no evidence of interarterial compression.

**Figure 1:**
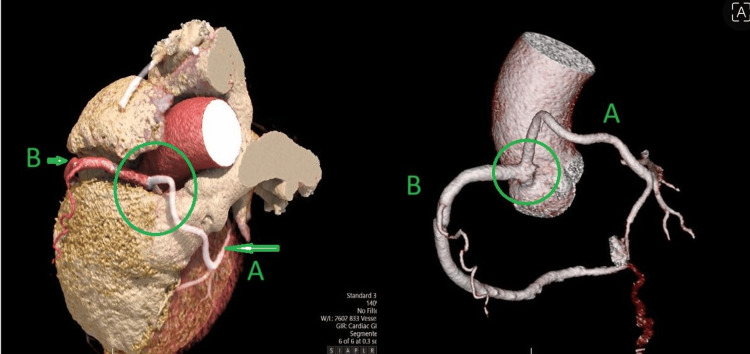
Coronary Computed Tomography Angiography (CCTA) Demonstrating Anomalous Origin of the Left Main Coronary Artery (LMCA) From the Proximal Right Coronary Artery (RCA). The LMCA Follows a Prepulmonic (Anterior) Course Without Interarterial Compression. (A) Left Main Coronary Artery. (B) Right Coronary Artery.

The coronary artery calcium (CAC) score was zero, and no obstructive atherosclerotic plaque was identified. Based on Lipton’s angiographic classification, this anomaly was categorized as a RIIA type single coronary artery.

Given the benign anatomical course, absence of high-risk imaging features, normal biomarkers, and low clinical suspicion for ischemia, conservative management with clinical surveillance and cardiovascular risk factor optimization was recommended. Additional functional ischemia testing was deferred initially and reserved for any progression to exertional or persistent symptoms.

## Discussion

Coronary artery anomalies (CAAs) are uncommon congenital abnormalities that range from incidental benign variants to clinically significant lesions associated with myocardial ischemia and sudden cardiac death (SCD). The reported incidence of CAAs varies from approximately 0.2% to 1.3% among patients undergoing coronary angiography and approximately 0.3% in autopsy series [1,2]. Single coronary artery (SCA) anomalies are particularly rare, with an incidence of approximately 0.06% in large angiographic cohorts [5].

Common anomalous coronary origins include the left circumflex artery arising from the right coronary sinus or right coronary artery (RCA), the RCA arising from the left coronary sinus, and less frequently the left main coronary artery (LMCA) arising from the right coronary sinus [3]. Lipton and colleagues proposed an angiographic classification system for SCA based on ostial origin, anatomical distribution, and the course of the transverse trunk [5]. The Lipton et al. classification is summarized in Table 2.

**Table 2:** Lipton et al. Classification of Single Coronary Artery (SCA). Table 2 is created by the authors. It is not a verbatim copy of the source [5].

Classification Component	Code	Meaning
Site of origin	R	Origin from right coronary sinus
	L	Origin from left coronary sinus
Branching pattern	I	Single artery follows the course of either normal RCA or LCA
	II	One main coronary artery gives rise to the other from proximal segment
	III	LAD and LCx arise separately from proximal RCA
Course of transverse trunk	A	Anterior (prepulmonic) to pulmonary artery
	B	Interarterial (between aorta and pulmonary artery)
	P	Posterior (retroaortic) to aorta
	S	Septal (subpulmonic/intramyocardial)
	C	Combined or complex course

In this classification, RIIA denotes origin from the right coronary sinus (R), early branching where one coronary system gives rise to the other (Type II), and an anterior/prepulmonic course (A) relative to the pulmonary artery [5]. Accordingly, our case is classified as RIIA, a pattern generally associated with a benign clinical course when no high-risk anatomical features are present.

Anomalous origin of the LMCA from the right coronary sinus/RCA is exceptionally rare, with prevalence estimates ranging from 0.0024% to 0.02% [1,5]. Yamanaka and Hobbs reported the anomalous origin of the left coronary artery from the right sinus as one of the rarest coronary anomalies in large angiographic series [1]. In our patient, the LMCA originated specifically from the proximal RCA, a variant that appears even less frequently described.

Although CAAs are uncommon, they represent an important substrate for adverse cardiac events, particularly in younger individuals and athletic populations [3,4]. However, extrapolation of these risk estimates to older adults should be undertaken cautiously, as age-related vascular compliance changes, acquired atherosclerotic burden, comorbid conditions, and symptom characteristics may significantly influence clinical presentation and prognosis.

The clinical risk associated with coronary artery anomalies is primarily determined by the anatomical course of the anomalous vessel rather than the site of origin alone (Table 3).

**Table 3:** Clinical Risk Stratification Based on Anatomical Course of Anomalous Coronary Arteries. SCD: sudden cardiac death

Anatomical Course	Description	Mechanism of Ischemia / SCD	Clinical Risk	Key References
Interarterial	Courses between the aorta and pulmonary artery	Slit-like ostium, acute take-off angle, intramural segment, dynamic compression during exertion	High risk (associated with SCD)	[3,7]
Prepulmonic (Anterior)	Courses anterior to the pulmonary artery	No compression between great vessels	Generally benign	[8,9]
Retroaortic	Courses posterior to the aortic root	No dynamic compression	Benign	[8]
Transseptal (Subpulmonic)	Intramyocardial or septal course beneath pulmonary artery	Rarely associated with ischemia	Usually benign	[8,9]

Interarterial variants, coursing between the aorta and pulmonary artery, carry the highest risk of ischemia and SCD [3,7]. Proposed mechanisms include slit-like ostium, acute takeoff angle, intramural aortic segments, endothelial injury, and dynamic compression during exertion [3,7]. In selected autopsy-based series, the risk of sudden death in high-risk anomalous coronary patterns has been reported to be substantial [12]. In contrast, prepulmonic (anterior), retroaortic, and transseptal courses are typically considered benign variants with favorable prognoses [8,9].

Accurate delineation of coronary origin and course is therefore essential for risk stratification and management. While invasive coronary angiography remains valuable for evaluating luminal disease, it can be limited in defining the three-dimensional course of anomalous vessels [11]. Coronary computed tomography angiography (CCTA) provides high-resolution three-dimensional assessment and is widely regarded as the preferred modality for anatomical characterization of CAAs [8,11,13]. In our case, CCTA clearly demonstrated a prepulmonic course without interarterial compression or intramural segment and no obstructive atherosclerosis (CAC score of 0), supporting a low-risk anatomical profile.

Management of coronary artery anomalies depends on symptoms, objective evidence of ischemia, and anatomical risk characteristics. High-risk interarterial variants may warrant surgical correction, whereas lower-risk variants without demonstrable ischemia are often managed conservatively with clinical surveillance and cardiovascular risk factor optimization [3,4]. Current eligibility recommendations also advise restriction from competitive athletics for selected untreated high-risk anomalous aortic origin patterns [4].

In the present case, conservative management was selected based on an integrated low-risk profile that included atypical non-exertional symptoms, normal cardiac biomarkers, absence of obstructive atherosclerotic disease, zero coronary calcium score, and a benign prepulmonic course without high-risk anatomical features. Although functional ischemia testing such as stress imaging or CT-derived fractional flow reserve may offer additional physiological assessment in selected symptomatic patients, these modalities were deferred given the low immediate suspicion for flow-limiting ischemia and the patient’s subsequent stable clinical course. We acknowledge that observations from a single case with three-year follow-up are hypothesis-generating and should not be interpreted as sufficient to alter standardized management guidelines for this rare RIIA variant. Rather, this case supports the importance of individualized decision-making based on anatomy, symptoms, and longitudinal follow-up.

## Conclusions

We report a rare case of single coronary artery anomaly involving anomalous origin of the left main coronary artery (LMCA) from the proximal right coronary artery (RCA) with a prepulmonic course. Although coronary artery anomalies may be associated with adverse outcomes, risk varies according to anatomical course, associated high-risk features, and patient-specific factors.

This case highlights the value of coronary computed tomography angiography in defining coronary anatomy and guiding individualized risk assessment. However, anatomical findings alone may not fully establish physiological significance, and adjunctive functional testing may be considered when symptoms persist or uncertainty remains.

Given the favorable three-year follow-up in this patient, conservative management was reasonable in this clinical context. Nevertheless, conclusions from a single case should be interpreted cautiously. Surveillance strategies for lower-risk variants remain individualized, and further long-term data are needed to better define optimal management of rare coronary anomalies.

## References

[1] Yamanaka O, Hobbs RE (1990). Coronary artery anomalies in 126,595 patients undergoing coronary arteriography. Cathet Cardiovasc Diagn.

[2] Baltaxe HA, Wixson D (1977). The incidence of congenital anomalies of the coronary arteries in the adult population. Radiology.

[3] Angelini P (2002). Coronary artery anomalies--current clinical issues: definitions, classification, incidence, clinical relevance, and treatment guidelines. Tex Heart Inst J.

[4] Maron BJ, Zipes DP (2005). Introduction: eligibility recommendations for competitive athletes with cardiovascular abnormalities-general considerations. J Am Coll Cardiol.

[5] Lipton MJ, Barry WH, Obrez I (1979). Isolated single coronary artery: diagnosis, angiographic classification, and clinical significance. Radiology.

[6] Ghersin E, Litmanovich D, Ofer A (2004). Anomalous origin of right coronary artery: diagnosis and dynamic evaluation with multidetector computed tomography. J Comput Assist Tomogr.

[7] Angelini P (2007). Coronary artery anomalies: an entity in search of an identity. Circulation.

[8] Villa AD, Sammut E, Nair A (2016). Coronary artery anomalies overview: The normal and the abnormal. World J Radiol.

[9] Young PM, Gerber TC, Williamson EE (2011). Cardiac imaging: Part 2, normal, variant, and anomalous configurations of the coronary vasculature. AJR Am J Roentgenol.

[10] Kilic A, Kilic A, Sultan I (2018). Anomalous origin of the left main coronary artery from the right coronary artery. Circ Cardiovasc Imaging.

[11] Ghadri JR, Kazakauskaite E, Braunschweig S (2014). Congenital coronary anomalies detected by coronary computed tomography compared to invasive coronary angiography. BMC Cardiovasc Disord.

[12] Lipsett J, Cohle SD, Berry PJ (1994). Anomalous coronary arteries: a multicenter pediatric autopsy study. Pediatr Pathol.

[13] Flessas D, Mamarelis I, Maniatis V (2013). An unusual pattern of three major components of the cardiovascular system: multimodality imaging and review of the literature. J Cardiothorac Surg.

